# Molecular Basis of the Divergent Immunogenicity of Two Pediatric Tick-Borne Encephalitis Virus Vaccines

**DOI:** 10.1128/JVI.02985-15

**Published:** 2016-01-28

**Authors:** Yvonne Beck, Richard Fritz, Klaus Orlinger, Stefan Kiermayr, Reinhard Ilk, Daniel Portsmouth, Eva-Maria Pöllabauer, Alexandra Löw-Baselli, Annett Hessel, Doris Kölch, M. Keith Howard, P. Noel Barrett, Thomas R. Kreil

**Affiliations:** a Vaccine R&D, Baxter Bioscience, Orth an der Donau, Austria; b Global Quality, Baxter Bioscience, Orth an der Donau, Austria; c Vaccine R&D, Baxter Bioscience, Vienna, Austria; d Pfizer Biotech, Dublin, Ireland; e Pfizer Manufacturing Austria, Orth an der Donau, Austria

## Abstract

Studies evaluating the immunogenicity of two pediatric tick-borne encephalitis virus (TBEV) vaccines have reported contradictory results. These vaccines are based on two different strains of the European TBEV subtype: FSME-Immun Junior is based on the Neudörfl (Nd) strain, whereas Encepur Children is based on the Karlsruhe (K23) strain. The antibody (Ab) response induced by these two vaccines might be influenced by antigenic differences in the envelope (E) protein, which is the major target of neutralizing antibodies. We used an established hybrid virus assay platform to compare the levels of induction of neutralizing antibodies against the two vaccine virus strains in children aged 1 to 11 years who received two immunizations with FSME-Immun Junior or Encepur Children. The influence of amino acid differences between the E proteins of the Nd and K23 vaccine strains was investigated by mutational analyses and three-dimensional computer modeling. FSME-Immun Junior induced 100% seropositivity and similar neutralizing antibody titers against hybrid viruses containing the TBEV E protein of the two vaccine strains. Encepur Children induced 100% seropositivity only against the hybrid virus containing the E protein of the homologous K23 vaccine strain. Antibody responses induced by Encepur Children to the hybrid virus containing the E protein of the heterologous Nd strain were substantially and significantly (*P* < 0.001) lower than those to the K23 vaccine strain hybrid virus. Structure-based mutational analyses of the TBEV E protein indicated that this is due to a mutation in the DI-DII hinge region of the K23 vaccine strain E protein which may have occurred during production of the vaccine seed virus and which is not present in any wild-type TBE viruses.

**IMPORTANCE** Our data suggest that there are major differences in the abilities of two European subtype pediatric TBEV vaccines to induce antibodies capable of neutralizing heterologous TBEV strains. This is a result of a mutation in the DI-DII hinge region of the E protein of the K23 vaccine virus strain used to manufacture Encepur Children which is not present in the Nd strain used to manufacture FSME-Immun Junior or in any other known naturally occurring TBEVs.

## INTRODUCTION

Tick-borne encephalitis virus (TBEV) is a major human-pathogenic flavivirus that is endemic in Europe and Asia (1). Infection with TBEV can result in fatality or serious long-term neurological sequelae (1, 2). Licensed inactivated whole-virus TBEV vaccines are available from two European manufacturers, FSME-Immun (Pfizer Corporation, Vienna, Austria) (3–6) and Encepur (Novartis Vaccines and Diagnostics, Marburg, Germany) (7, 8), and are based on European subtype TBEV strains Neudoerfl (Nd) and Karlsruhe (K23), respectively. For children aged 1 to 11 years, both vaccines are available in pediatric formulations (FSME-Immun Junior and Encepur Children) (2, 6, 7). The pediatric versions of FSME-Immun Junior and Encepur Children are identical to the adult vaccine, the only differences being the doses, 0.25 ml and 0.5 ml, respectively. The conventional primary vaccination schedules for these vaccines consist of three doses administered at 0, 1 to 3, and 5 to 12 months for FSME-Immun or at 0, 1 to 3, and 9 to 12 months for Encepur (2). Vaccination is highly effective (9), and the incidence of TBE has decreased substantially in regions of TBEV infection endemicity with successful vaccination programs (2). There is a highly significant correlation between vaccine-induced virus-neutralizing antibody titers and IgG antibody titers, which correlate with protection against TBE (10, 11).

FSME-Immun and Encepur have both been shown to induce high rates of neutralizing antibody seropositivity in clinical studies in adults (3, 4, 8) and children (6, 7). However, comparative immunogenicity evaluations in children have given contradictory results. One study reported that two immunizations with FSME-Immun Junior induced higher neutralizing antibody titers against the Nd virus strain than did immunizations with Encepur Children (6). In contrast, a second study reported that two immunizations with Encepur Children induced higher rates of neutralizing antibodies against the K23 vaccine strain virus than did immunizations with FSME-Immun Junior. However, this difference was significantly reduced when the Nd virus rather than the K23 vaccine strain virus was used to measure neutralizing antibody titers (12).

The mechanism(s) responsible for the reported differences in the abilities of FSME-Immun and Encepur to induce neutralizing antibodies against different TBEV strains has not previously been analyzed in detail. Antigenic differences in the envelope (E) protein, the major target of neutralizing antibodies, of the two vaccine strains, Nd and K23, might influence the ability of vaccine-induced antibodies to neutralize heterologous TBEV strains. Analysis of the E protein sequences published for the Nd and original wild-type K23 field isolates reveals three amino acid differences at positions 83, 136, and 167 (13). In addition, it was recently reported that the K23 isolate used for manufacture of Encepur contains an additional substitution at position 52 of the E protein (14) (GenBank accession no. AM600965.1) which is not present in the original K23 field isolate (GenBank accession no. AF091010.1). In contrast to the naturally occurring amino acid differences in the E proteins, the mutation at position 52 of the E protein in the Encepur vaccine strain is located in the DI-DII hinge region connecting E protein domains DI and DII (15). For a number of flaviviruses, virus neutralizing antibodies have been identified which locate to the DI-DII hinge region (16–18).

In addition to potential antigenic differences between the Nd and K23 vaccine viruses, different infectivity and replicative capacities of these TBEVs in cell cultures used for virus neutralization assays might also have contributed to the contradictory study results reported previously. To overcome this limitation associated with classical TBEV neutralization assays, we previously established an assay platform, based on hybrid viruses that share the West Nile virus (WNV) backbone and encode the individual TBEV membrane and envelope proteins, that enables analysis of virus neutralization in a setting that equalizes virus replication and infectivity levels while maintaining the individual virus surface characteristics that determine virus neutralization (19). In the present study, we utilized this established assay system to compare the abilities of FSME-Immun Junior and Encepur Children to induce neutralizing antibodies against hybrid viruses containing the E proteins of the two TBEV vaccine virus strains, Nd and K23. We also investigated the impact of amino acid differences in the E proteins of the Nd and K23 vaccine virus strains on their ability to induce cross-neutralizing antibody responses.

## MATERIALS AND METHODS

### Clinical trial and vaccines.

Serum samples were derived from a multicenter phase III study (EUDRACT 2008-002691-10) in 301 children aged 1 to 11 years (6). Participants were stratified by age (1 to 2 years, 3 to 6 years, and 7 to 11 years) and randomized 1:1 to receive two immunizations, 4 weeks apart, with FSME-Immun Junior (lot no. VNR1H04E) or Encepur Children (lot no. 091021A). Blood for serological analysis was drawn before and 28 days after each vaccination. Written informed consent was obtained for all participants prior to enrollment. Prior Institutional Review Board (IRB) approval was obtained from each institution that participated in the research.

### Hybrid WNVs/TBEVs used for neutralization assays.

The generation of hybrid West Nile viruses (WNVs)/TBEVs expressing the precursor of the membrane (prM) and E proteins of TBEV on a WNV backbone has been described previously (19). Briefly, nucleotide sequences of prM and E proteins of TBEV strain Nd (GenBank accession no. U27495.1), and of the K23 strain used for Encepur manufacture (GenBank accession no. AM600965), flanked by WNV NY99 (GenBank accession no. AF196835) coding sequences, were generated by chemical synthesis (GeneArt) and cloned into a previously reported WNV cDNA system (20). In the hybrid viruses, the WNV prM and E sequences were exchanged with the respective TBEV sequences, which were embedded in the WNV backbone starting at the NS2B3 cleavage site of the capsid protein and ending at the signalase cleavage site of the NS1 signal sequence, such that the complete sequences of TBEV prM and E and the respective signal sequences were preserved, allowing efficient viral assembly.

In addition, single-point mutations were introduced into the Nd hybrid virus E protein by PCR, using the GeneArt site-directed mutagenesis system, at amino acid positions 52, 83, 136, and 167, resulting in hybrid viruses carrying N52K, A83T, K136R, and I167V substitutions in the Nd E protein. Using the same method, a single-point mutation at position 52 of K23 E protein was introduced, resulting in a hybrid virus carrying a K52N substitution in the K23 E protein.

*In vitro* transcription and transfection of Vero cells yielded the Nd and K23 infectious hybrid viruses expressing Nd and the K23 prM and E proteins, respectively. To confirm the expected phenotype of the hybrid viruses, the presence of heterologous TBEV protein E on hybrid virus particles was analyzed by Western blotting using polyclonal anti-TBEV Neudörfl and anti-WNV NY99 antisera, as previously described (19). Hybrid viruses recovered from transfected cells were sequenced to demonstrate that no reversions or additional mutations occurred in the viruses used in subsequent neutralization assays.

To verify that there were no significant differences between hybrid viruses with respect to infectious titers and particle-to-infectivity ratios (which determine virus behavior in neutralization assays), the levels of infectivity of the individual hybrid viruses versus the levels of genome-containing viral particles exported into the cell supernatant were determined by quantitative PCR (qPCR) and viral 50% tissue culture infective dose (TCID_50_) titer analysis as previously described (19).

To verify that no changes in the overall conformation of the E protein had occurred, hybrid viruses containing single-point mutations in the E protein were compared to Nd wild-type virus by a four-layer enzyme-linked immunosorbent assay (ELISA)-based epitope mapping method, using a set of E protein-specific monoclonal Abs (MAbs), as previously described (21). These monoclonal antibodies react with the neutral pH form of the TBEV E protein, whose binding sites have been mapped by the use of neutralization-escape mutants as well as specifically engineered mutations. An advantage of this assay is that the antigen under investigation is not subject to the denaturation effects that occur during the coating of proteins onto solid phases, which would impact the antigenic structure of the E protein (21). These investigations confirmed the lack of conformational change in the E protein upon introduction of the individual amino acid substitutions (results not shown).

### Virus titration and growth curves.

A549 cells grown in 175-cm^2^ tissue culture flasks were infected with TBEV hybrids at a multiplicity of infection of 0.0001. After incubation for 1 h, the inoculum was removed, the cells were washed with 10 ml Dulbecco's modified Eagle's medium, and 75 ml of fresh medium was added. At various time points (1, 6, 24, 48, 54, 72, and 96 h) after infection, 1 ml of culture medium was removed and the virus titer determined by TCID_50_ assay, as described elsewhere (19). The maximum deviation of the assay was ±1 log.

### Virus neutralization assays.

Virus neutralization assays utilizing WNV/TBEV hybrid Nd and K23 viruses were carried out in A549 cells, in which the replication kinetics and titers of hybrid viruses and parent TBEVs are highly similar, as previously described (19). Briefly, sera were complement inactivated by incubation at 56°C for 30 min, serially diluted, in 2-fold steps, in Dulbecco's modified Eagle's medium, and incubated at room temperature for 1 h ± 10 min with an equal volume of individual hybrid viruses, such that the virus concentration in the assay was 2 × 10^3^ TCID_50_/ml. Samples were then transferred to 96-well microtiter plates preseeded with A549 cells. Cells were assessed for the presence of cytopathogenic effects after incubation at 37°C and 5% CO_2_ for 7 days ± 1 day. This assay is fully validated with respect to repeatability, precision, specificity, linearity, range, and robustness, in accordance with ICH topic Q2 (R1) (“Validation of Analytical Procedures: Text and Methodology”). The maximum deviation of assay repeatability and intermediate precision was shown to be less than 2-fold of the mean value. The assay validation was performed with wild-type Nd virus. Virus neutralization titers were calculated according to the statistical approximation method of Spearman-Kärber. Subjects with virus neutralization titers of ≥1:7.7 (the limit of detection of the assay) were defined as seropositive. For the purposes of statistical analyses, titers below 1:7.7 were assigned a nominal titer of 1:3.9.

### *In silico* and statistical analysis.

Three-dimensional modeling of the TBEV strain Neudörfl E protein was done using Pymol software. Comparisons of neutralizing antibody titers were done by one-factorial analysis of variance (ANOVA) and the Tukey test using validated Minitab v15.0 software, with the underlying assumption that the factors in the nested hierarchy (choice of individuals, measurement data) were random. The sensitivity of the study design to determine the effect of amino acid substitutions in the TBEV E protein on the detection of neutralizing antibodies induced by Encepur Children was assessed by performing a power analysis, whereby the group size *n* was uniformly aligned with the smallest groups. Confidence intervals were calculated on a log scale to account for the log-normal distribution of residuals.

## RESULTS

### Neutralization of hybrid viruses expressing the surface proteins from TBEV strains used to manufacture FSME-Immun and Encepur.

**FIG 1:**
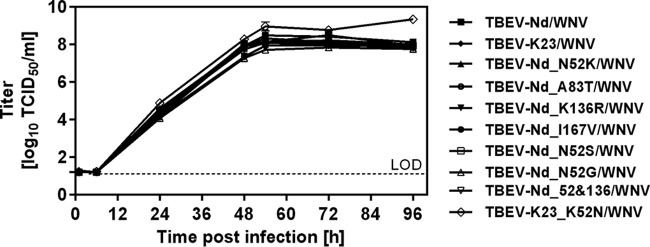
Replication kinetics of hybrid TBEVs/WNVs in cell culture. Viral growth was analyzed in A549 cells infected at a multiplicity of infection (MOI) of 0.0001. Samples collected at the indicated time points postinfection were analyzed by the TCID_50_ method based on A549 cells. The tests were performed at least twice. Variability of the assay is ±1 log. LOD, limit of detection.

To evaluate the ability of FSME-Immun and Encepur to induce antibodies neutralizing the respective TBEV strains used for vaccine manufacture, i.e., wild-type Nd and the K23 vaccine strain, a head-to-head study was carried out. This was done using an established neutralization assay which utilizes hybrid WNVs/TBEVs expressing the surface prM and E proteins of TBEV (19). Because differences in the growth kinetics and stability of the different hybrid viruses utilized in this investigation could influence the neutralizing antibody titers detected by each hybrid virus, it was important to demonstrate that all of the hybrid viruses used in the investigation had the same growth kinetics. These data are presented in Fig. 1 and confirm that the growth kinetics of all hybrid viruses were highly similar.

The ability of FSME-Immun and Encepur to induce antibodies neutralizing the hybrid viruses displaying the surface proteins of either the Nd strain or the K23 vaccine strain was investigated using immune sera from a comparative study (6) where children aged 1 to 11 years received two immunizations with either FSME-Immun Junior (*n* = 149) or Encepur Children (*n* = 152).

**FIG 2:**
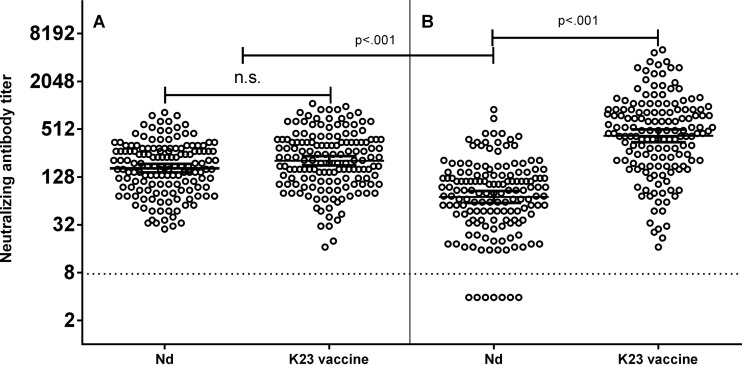
Neutralizing antibody titers induced by two immunizations with (A) FSME-Immun Junior and (B) Encepur Children in participants aged 1 to 11 years. Data in each panel are individual neutralizing antibody titers, geometric mean titers (GMT) (wide horizontal line), and 95% confidence intervals (CI) (narrow horizontal lines) against hybrid viruses displaying the surface proteins of TBEV strain Nd or the Encepur K23 vaccine strain. The horizontal dotted line represents the limit of detection of the assay. n.s., nonsignificant.

Figure 2 shows individual neutralizing antibody titers and geometric mean titers (GMTs) induced in study participants against the Nd and K23 vaccine strain hybrid viruses by FSME-Immun Junior (Fig. 2A) and Encepur Children (Fig. 2B). In participants receiving FSME-Immun Junior, antibody responses to both the Nd hybrid virus (GMT, 165; 95% confidence interval [CI], 146 to 186) and the K23 vaccine strain hybrid virus (GMT, 205; 95% CI, 179 to 235) were similarly high, and all participants achieved seropositivity against both hybrid viruses. In contrast, in participants immunized with Encepur Children, there was a striking difference in the responses to the two hybrid viruses. Antibody responses to the Nd hybrid virus (GMT, 72; 95% CI, 60 to 85) were substantially and significantly (*P* < 0.001) lower than those to the K23 vaccine strain hybrid virus (GMT, 418; 95% CI, 347 to 503). Moreover, neutralizing antibody responses to the Nd hybrid virus induced by Encepur Children were significantly (*P* < 0.001) lower than those induced by FSME-Immun Junior. Seven children (4.6%) immunized with Encepur Children did not achieve seropositivity against the Nd hybrid virus.

Table 1 shows the same analyses presented as GMTs, seropositivity rates, and ratios of GMTs against the Nd hybrid virus compared to the K23 vaccine strain hybrid virus, stratified by age group. These data show that the conclusions for the entire study population are also valid for each age group. In recipients of FSME-Immun Junior, there were no significant differences in GMT or seropositivity rates against the Nd or K23 vaccine strain hybrid viruses in any age group. In contrast, neutralizing antibody titers induced in participants immunized with Encepur Children were 4.6-fold to 7.3-fold lower against the Nd hybrid virus than against the K23 vaccine strain hybrid virus in the different age groups (*P* < 0.001).

**TABLE 1:** Rates of seropositivity and GMTs induced against Nd and K23 vaccine strain viruses after two immunizations with FSME-Immun Junior or Encepur Children, stratified by age group

Age (yrs)	Vaccine	No. of participants	Seropositivity rate (%) against^*a*^:		GMT (95% CI) against:		Ratio of GMTs against K23 vaccine strain and Nd viruses
			Nd virus	K23 vaccine strain virus	Nd virus	K23 vaccine strain virus	
1–2	FSME-Immun Junior	50	100	100	158 (126–199)	176 (137–226)	1.1
	Encepur Children	50	94	100	52 (39–68)	379 (287–501)	7.3
3–6	FSME-Immun Junior	51	100	100	194 (156–240)	260 (204–332)	1.3
	Encepur Children	51	96.1	100	89 (64–124)	529 (367–761)	5.9
7–11	FSME-Immun Junior	48	100	100	146 (120–178)	186 (149–233)	1.3
	Encepur Children	51	96.1	100	79 (59–107)	364 (261–508)	4.6

a Subjects with virus neutralization titers of ≥1:7.7 (the limit of detection of the assay) were defined as seropositive.

^b^ GMT, geometric mean titer; CI, confidence interval.

Table 2 shows the proportions of Encepur Children and FSME-Immun Junior recipients who had higher neutralizing antibody titers against the K23 vaccine strain hybrid virus than against the Nd hybrid virus. Strikingly, the antibody response in Encepur Children recipients was highly skewed toward the K23 vaccine strain, with 96% of the sera from Encepur Children recipients having a greater-than-2-fold-higher titer against the K23 vaccine strain hybrid virus and 78.3% of subjects having a greater-than-4-fold-higher titer against the K23 vaccine strain hybrid virus. In contrast, the responses to the Nd virus and K23 vaccine strain virus were highly similar in FSME-Immun Junior recipients.

**TABLE 2:** Fold differences in neutralizing antibody titers against the Nd and K23 vaccine strain hybrid viruses

Fold difference in K23:Nd neutralizing antibody titers	No. (%) of subjects with indicated K23:Nd titers receiving:	
	FSME-Immun Junior	Encepur Children
>4	1/149 (0.7)	119/152 (78.3)
>2	18/149 (12)	146/152 (96)
≤2	131/149 (87)	6/152 (3.9)

### Identification of an amino acid in the TBEV envelope protein critical for the induction of cross-neutralizing antibody responses.

We next aimed at investigating the possibility that the different abilities of FSME-Immun and Encepur to induce cross-neutralizing antibody responses might be a consequence of amino acid differences in the E proteins of the Nd and K23 vaccine strains. To this end, mutant Nd hybrid viruses were generated by substituting, individually, each of the four E protein amino acids which differ between the Nd and K23 vaccine strains. Three of these amino acid substitutions, i.e., the A83T, K136R, and I167V substitutions (Table 3), are based on the published sequences (13) of the original wild-type Nd and K23 virus isolates. A fourth substitution, N52K, was reported only for the K23 vaccine strain (14).

**TABLE 3:** E protein amino acid differences between Nd, wild-type K23 and K23 vaccine strain viruses

Virus	E protein amino acid at position:			
	52	83	136	167
Nd (FSME-Immun)	N	A	K	I
K23 wild type	N	T	R	V
K23 vaccine strain (Encepur)	K	T	R	V

N, asparagine; A, alanine; K, lysine; I, isoleucine; T, threonine; R, arginine; V, valine.

**FIG 3:**
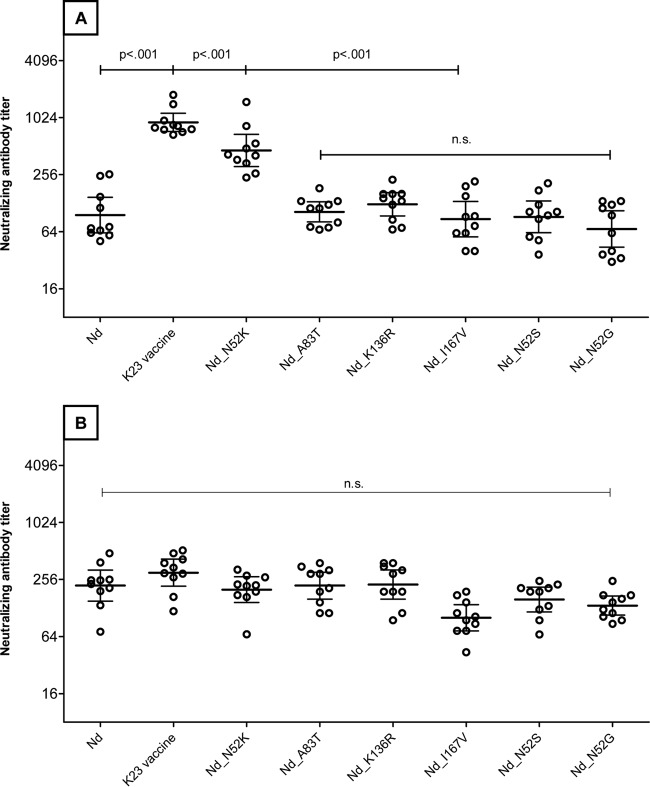
Effect of amino acid substitutions in the Neudörfl strain TBEV envelope protein on the ability of hybrid viruses to detect neutralizing antibodies induced by Encepur Children and FSME-Immun Junior. Ten sera from children immunized with Encepur Children (A) and 10 from children immunized with FSME-Immun Junior (B) were selected which had low neutralizing antibody titers against the Nd virus but high titers against the K23 vaccine strain virus. Data in each panel are individual neutralizing antibody titers, geometric mean titers (GMT) (wide horizontal line), and 95% confidence intervals (CI) (narrow horizontal lines) against hybrid viruses displaying the surface proteins of TBEV strain Nd or against hybrid viruses displaying Nd E protein containing N52K, A83T, K136R, I167V, N52S, and N52G substitutions. GMT ratios are calculated compared to the Nd strain. n.s., nonsignificant.

To allow good resolution of amino acid substitutions which influence the detection of neutralizing antibodies, 10 immune sera were chosen from participants immunized with Encepur Children which all had high neutralizing antibody titers against the K23 vaccine strain hybrid virus but low titers against the Nd hybrid virus. Each of these sera was used to measure neutralizing antibody titers against the panel of mutant Nd hybrid viruses. Figure 3A shows the individual neutralizing antibody titers and GMTs for these 10 antisera. Neutralizing antibody titers against the A83T, K136R, and I167V mutant Nd hybrid viruses (GMT range, 87 to 125) were not significantly different from those against the wild-type Nd hybrid virus (GMT, 96) but were significantly (*P* < 0.001) lower than those against the K23 vaccine strain hybrid virus (GMT, 911), indicating that these substitutions do not independently influence the capacity of the TBEV hybrid viruses to be neutralized by antibodies induced by Encepur Children. In contrast, the N52K substitution resulted in substantially increased neutralizing antibody titers (GMT, 461), which, although significantly lower than the titers obtained with the K23 vaccine strain hybrid, were still significantly higher than the titers against the Nd and other Nd mutant hybrid viruses. These data demonstrate the importance of position 52 of the TBE E protein for virus neutralization.

To assess the potential impact of the N52K mutation on the ability of Encepur to induce neutralizing antibodies against other naturally occurring TBEV strains, we analyzed the E protein sequences of published TBEVs. Protein sequence analysis of the E proteins of >250 naturally occurring TBEVs showed that the majority of viruses, including the Nd strain, contain asparagine at position 52 and that a small number of viruses contain serine or glycine (data not shown). Asparagine, serine, and glycine are all small, uncharged amino acids. In contrast, the lysine in position 52 of the K23 vaccine strain is a large, positively charged amino acid.

We therefore also analyzed mutant hybrid viruses containing either a serine or glycine in position 52 (N52S and N52G substitutions), thus accounting for all amino acid variations at position 52 which have been reported for naturally occurring TBEV strains. Interestingly, both the N52S and N52G substitutions resulted in titers of neutralizing antibodies in Encepur antisera (Fig. 3A) that were as low as those of the Nd mutant hybrid strains. These additional data support a hypothesis that the N52K mutation might affect the ability of Encepur to induce cross-neutralizing antibodies.

To verify that this phenomenon is specific to Encepur, we investigated the ability of 10 FSME-Immun sera to neutralize the panel of hybrid viruses. To allow good comparability with the 10 sera tested from the Encepur Children recipients, we selected 10 FSME Immun Junior sera which also had relatively high titers against the K23 virus compared to the Nd virus. The data in Fig. 3B demonstrate that there are no statistically significant differences in the antibody responses to any of the panel of hybrid viruses in the sera from FSME Immun Junior recipients.

**FIG 4:**
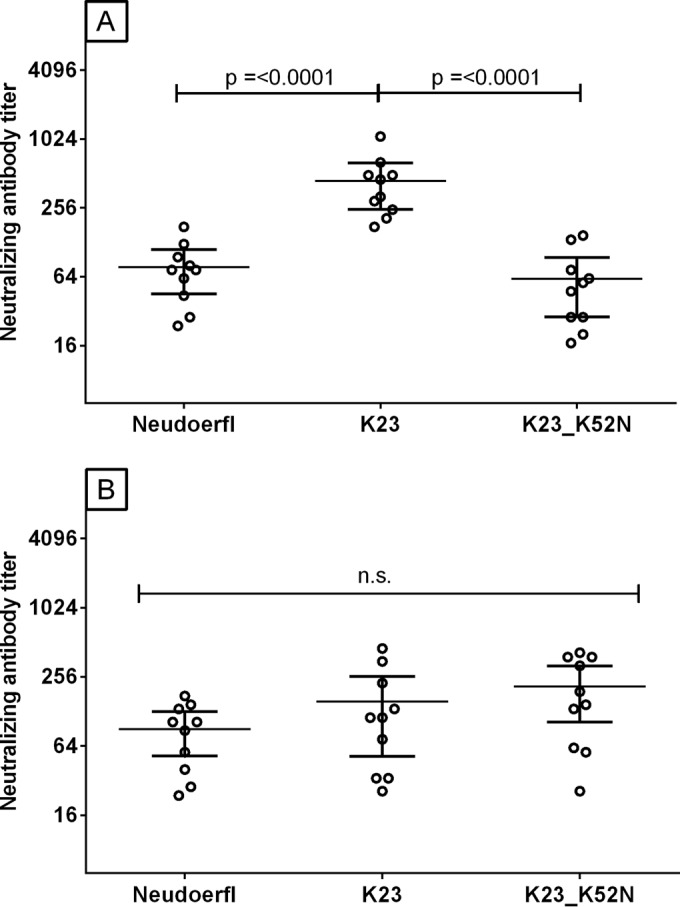
Effect of amino acid substitutions in the K23 strain TBEV envelope protein on the ability of hybrid viruses to detect neutralizing antibodies induced by Encepur Children and FSME-Immun Junior. Ten sera from children immunized with Encepur Children (A) and 10 from children immunized with FSME-Immun Junior (B) were selected which had low neutralizing antibody titers against the Nd virus but high titers against the K23 vaccine strain virus. Data in each panel are individual neutralizing antibody titers, geometric mean titers (GMT) (wide horizontal line), and 95% confidence intervals (CI) (narrow horizontal lines) against hybrid viruses displaying the surface proteins of TBEV strain Nd, the K23 vaccine strain, or the K23 vaccine strain containing the K52N substitutions. GMT ratios are calculated compared to the K23 strain. n.s., nonsignificant.

To confirm that the elevated neutralizing antibody titers obtained in assays performed with the K23 vaccine strain and the Nd hybrid having the N52K mutation are solely dependent on the presence of a lysine residue at position 52 of the E-protein, a hybrid virus based on the K23 vaccine strain possessing a reciprocal K52N mutation was constructed. Figure 4A shows the individual neutralizing antibody titers and GMTs for 10 high-titer antisera of children immunized with Encepur Children tested with the Nd hybrid, K23 vaccine hybrid, and K23-K52N hybrid. The results demonstrate that replacement of the lysine at amino acid position 52 in the K23 vaccine strain with asparagine causes an approximately 8-fold reduction in GMT (GMT, 38 to 48) to a titer similar to the titer obtained with the Nd hybrid (GMT, 66). Tests performed with 10 sera from FSME-Immun Junior resulted in neutralizing antibody titers against the Nd hybrid, K23 vaccine hybrid, and K23 K52N hybrid (GMT, 74 to 152) with equivalent levels of statistical significance.

**FIG 5:**
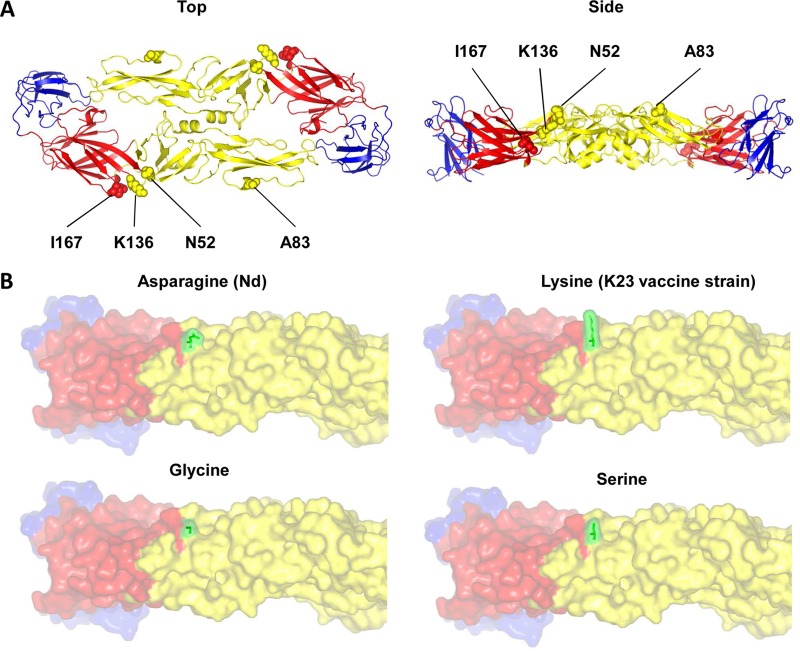
Influence of amino acid changes on the three-dimensional structure of the TBEV E protein. (A) Ribbon diagrams (top and side views) of the structure of the E ectodomain of strain Nd were generated using the PyMOL molecular visualization system (https://www.pymol.org/). The positions of amino acid variations with respect to the K23 vaccine strain are highlighted with spheres. Red, yellow, and blue coloring indicates E protein domains I, II, and III. (B) Three-dimensional models of TBEV strain Nd with asparagine, lysine, glycine, or serine in amino acid position 52 (green).

To predict the effect that the N52K substitution would have on the three-dimensional structure of the E protein (22), we used computer modeling to compare the predicted structures of E proteins containing asparagine, serine, glycine, and lysine at position 52. Figure 5 shows that the three small, uncharged amino acids found in all naturally occurring TBEV strains do not protrude from the surface of the E protein. In contrast, the large, positively charged lysine at position 52 of the K23 vaccine virus strain E protein is predicted to be out of plane and to protrude markedly from the E protein surface.

## DISCUSSION

We evaluated the abilities of two pediatric TBEV vaccines, FSME-Immun Junior and Encepur Children, to induce neutralizing antibodies against the respective TBEVs used for vaccine manufacture, i.e., wild-type Nd and vaccine strain K23, utilizing an established assay system comprising hybrid WNVs/TBEVs expressing the prM and E proteins of the respective vaccine virus strains. This strategy preserves the antigenic characteristics of the individual wild-type isolates while removing bias generated by differences in viral growth or infectivity in the cell-based virus neutralization assay system used (19). As previously demonstrated using this assay system in adults immunized with FSME-Immun (19), high titers of neutralizing antibodies were induced in children by FSME-Immun Junior against both the Nd and vaccine strain K23 hybrid viruses, with 100% of participants achieving seropositivity after two immunizations. In contrast, antibodies induced by Encepur Children showed a markedly different ability to neutralize the Nd and K23 vaccine hybrid viruses. Neutralizing antibody titers induced by Encepur Children against the Nd hybrid virus were substantially and significantly lower than against the K23 vaccine strain hybrid virus and were significantly lower than those induced by FSME-Immun Junior. Moreover, in a number of children immunized with Encepur Children, neutralizing antibodies against the Nd hybrid virus were not detectable. These data indicate that there are major differences in the abilities of the two vaccines to induce antibodies against heterologous TBEV strains.

Mutational analysis of the TBEV E protein suggests that the difference in the abilities of the two vaccines to induce cross-neutralizing antibody responses is a direct consequence of a single N52K amino acid substitution in the E protein of the K23 vaccine strain (14) that does not occur in the wild-type K23 strain or the Nd vaccine strain or in any other known naturally occurring TBEVs. We analyzed the E protein sequences of >250 published TBEV isolates (data not shown) and found that they all contained small, uncharged amino acids, such as asparagine (found in the Nd strain and the majority of other wild-type isolates), serine, or glycine, at position 52. In contrast, the lysine in position 52 of the K23 vaccine strain E protein is a large, positively charged amino acid residue that modeling predicted to protrude from the virus surface, as illustrated in Fig. 5. The reduced ability of the K23 vaccine strain to induce cross-neutralizing antibodies might thus be explained by the enhanced accessibility of the lysine residue compared to that seen with amino acids present in naturally occurring TBE viruses, such that neutralizing antibody induction is strongly skewed toward the N52K substitution in the E protein of the vaccine strain virus. This interpretation is supported by the observation that sera of individuals immunized with Encepur Children had significantly higher neutralizing antibody titers against both the K23 vaccine strain hybrid and the Nd N52K mutant hybrid (Fig. 3A), while sera of those immunized with FSME-Immun Junior showed no statistically significant differences in the antibody responses to any of the panel of hybrid viruses (Fig. 3B). The critical importance of this amino acid substitution at position 52 is further confirmed by the demonstration that the introduction of a K52N mutation into the K23 vaccine strain hybrid resulted in a significant drop in the neutralizing antibody titers of sera of individuals immunized with Encepur Children (Fig. 4A), while sera of those immunized with FSME-Immun Junior again showed no statistically significant differences in the antibody responses to any of the tested hybrid viruses (Fig. 4B).

Interestingly, many other flaviviruses also contain asparagine, serine, or glycine in position 52 of the E protein (15). Position 52 of TBEV E protein, in common with other flavivirus E proteins, is located in the hinge region connecting the E protein domains DI and DII (15). For a number of flaviviruses, including WNV, Japanese encephalitis virus, Murray Valley encephalitis virus, and dengue virus, virus neutralizing antibodies have been identified which locate to the DI-DII hinge region (16–18). For dengue virus, the DI-DII hinge region determines the serotype-specific neutralizing potency of human DENV immune sera, and data suggest that neutralizing antibodies which target the DI-DII hinge are a major component of protective immunity (16).

The N52K mutation in the E protein of the Encepur vaccine strain may have been acquired during the Encepur vaccine manufacturing process, prior to generation of the master seed bank. To investigate the stability of the Encepur K23 vaccine strain, Bröker et al. analyzed the nucleotide sequence encoding the E protein of the K23 virus from the master seed bank, the working seed bank, and a production lot and reported no sequence variation at these three stages of vaccine production (14). However, the original K23 virus isolate had already been subjected to substantial passaging in the brains of suckling (3 passages) and primary chicken embryo fibroblasts (19 passages) prior to generation of the master seed bank (14). This extensive passaging may have led to the acquisition of the critical N52K mutation present in the Encepur vaccine strain, since E protein sequence changes associated with culture adaptation of TBEV strains have been seen by other researchers (23).

In addition to the N52K substitution in the K23 vaccine strain, three naturally occurring E protein amino acid differences which are not located in the DI-DII hinge region exist between the Nd and K23 vaccine strains. Analysis of 10 sera from participants receiving Encepur Children performed using mutant Nd viruses containing the N52K substitution or the additional, naturally occurring substitutions indicated that the increased ability of the K23 vaccine strain to detect higher levels of neutralizing antibodies induced by Encepur Children is largely dependent on the N52K substitution. That this N52K substitution is sufficient for the higher level of neutralizing antibodies induced by Encepur Children was confirmed by tests of 10 sera from participants receiving Encepur Children performed using a mutant K23 vaccine strain virus containing the N52K substitution, which recreates the E protein of the original K23 isolate (14); the neutralizing antibody titers against the K23 K52N hybrid were found to be significantly lower than those against the K23 vaccine strain hybrid.

Our data suggest that the N52K substitution in the K23 vaccine strain results in an artificial epitope in a critical region for virus neutralization that is not present in any of the naturally occurring TBEV strains. Antibodies generated against this artificial epitope may be less relevant for the neutralization of wild-type TBEV strains present in the environment. Since Encepur induces neutralizing antibody titers that are significantly higher for the K23 vaccine strain than for any wild-type TBEV strain, assessing the immunogenicity of Encepur in a homologous system, using the K23 vaccine strain in the neutralization assay, might overestimate the efficacy of Encepur against naturally occurring TBEV strains. For a more relevant prediction of efficacy against circulating wild-type TBEV strains, the immune response induced by Encepur should be measured against wild-type TBEV strains and not against the artificial mutant K23 vaccine strain.
